# Healthcare Professionals Providing Direct Care to the COVID-19 Patients: A Descriptive Cross-sectional Study

**DOI:** 10.31729/jnma.6101

**Published:** 2022-02-28

**Authors:** Bishnu Dutta Acharya, Mangal Rawal, Dhiraj Gurung, Bhanu Limbu, Prem Laxmi Baniya, Poojan Kumar Rokya, Suresh Panthi

**Affiliations:** 1 Department of Physiotherapy, Karnali Academy of Health Sciences, Jumla, Nepal; 2 Department of Orthopaedic and Trauma, Karnali Academy of Health Sciences, Jumla, Nepal; 3 Department of Internal Medicine, Karnali Academy of Sciences, Jumla, Nepal; 4 United Nations World Food Programme, Lalitpur, Nepal; 5 Nepal Health Research Council, Ramshah Path, Kathmandu, Nepal; 6 Central Department of Management, Tribhuvan University, Kirtipur, Nepal

**Keywords:** *COVID-19*, *healthcare professionals*, *Nepal*, *risk*

## Abstract

**Introduction:**

COVID-19 highly contagious viruses that have reached every corner of the world. Despite the heroic efforts to control the pandemic, health care professional risk for COVID-19 exposure was an important measure to identify due to lack of personal protective equipment. The objective of the study was to find out the prevalence of healthcare professionals providing direct care to the COVID-19 patient.

**Methods:**

A descriptive cross-sectional was conducted through online questionnaire from June 2020 to August 2020. Ethical approval was obtained from the Ethical Review Board of Nepal Health Research Council (Reference number: 363/2020 P). World Health Organization risk assessment protocol questionnaire was used for COVID-19 and distributed among 300 health care workers of Nepal involved in the management of COVID-19 hospitals. Convenience sampling was used. The KoBo toolbox was used for online data collection. Data analysis was done using Statistical Package for the Social Sciences version 23. Point estimate at 95% Confidence Interval was calculated along with frequency and percentage for binary data.

**Results:**

Among 300 study participants, 109 (36.33%), (30.85-41.74 at 95% Confidence Interval) of participants provide direct care to infected patients. With total respondents, 41 (37.61%) were registered nurses, medical doctors 28 (25.68%) and paramedics 36 (33.02%).

**Conclusions:**

Health care workers who provide direct care to the COVID-19 patient were similar to other studies done in similar settings. About half of the participants were exposed to COVID-19 virus from the hospital setting rather than from community setting which is similar to the study done in similar setting which might be due to lack of follow of protocols during COVID-19 patient care.

## INTRODUCTION

COVID-19 is a rapidly emerging disease that has been classified as a pandemic by the World Health Organisation (WHO) with a robust capacity for human-to-human transmission, long incubation period, and asymptomatic infection.^12^ In Nepal, health care workers are working beyond their usual disciplinary boundaries resulting in work pressure by COVID-19.^2,3^ The virus is transmitted via close contact, lack of hygienic technique and fomites that survive in air.^4,5^

Nepalese health care professionals (HCP) were at high risk for the COVID-19 transmission due to a global shortage of Personal Protective Equipment (PPE), screening tools in hospitals, neglect of hygienic techniques due to workload in quarantine areas and security checkpoints.^6,7^ The occupied intensive care unit, isolation wards and quarantine with less number of health professionals hinder the focus on personal protection resulting in easy mode of transmission though they have hygienic awareness.^8^

The aim of this study was to find out the prevalence of HCP providing direct care to the COVID-19 patient.

## METHODS

A descriptive cross-sectional study was conducted among HCP from government-specified COVID-19 tertiary hospitals with duty stations at screening centres, COVID-19 hospitals, isolations, Intensive Care Units covering all provinces of Nepal. The study approval was obtained from the Ethical Review Board , Nepal Health Research Council (NHRC reference number: 363/2020 P). Data was collected through the online questionnaire from June 2020 to August 2020. To minimise the bias of direct care providers, the questionnaire was distributed to all selected HCP. HCP includes nurses, medical doctors, physiotherapists, medical laboratories, pharmacists, and radiologists working at inpatients, outpatients, COVID-19 ICU, isolation, laboratories, radiology department, emergency or in multi-departments.

Concerning HCP in Nepal according to Nepal Medical Council, Nepal Health Professional Council, and Nursing Council the study participants were calculated. The convenience sampling technique was used.

Sample size was calculated using the following formula:

n = Z^2^ × p × q / e^2^

  = (1.96)^2^ × 0.5 × (1-0.5) / (0.06)^2^

  = 267

Where,

n = minimum required sample size
Z = 1.96 at 95% Confidence Interval (CI)
p = prevalence taken as 50% for maximum sample size calculation
e = margin of error, 6%

Considering 10% non response rate, the sample size was calculated to 297. However, the total healh care workers taken was 300. Due to prolonged duty hours, the risk of using the mobile phone while working, limited access to the internet in rural areas, and limited time decrease the response rate than expected.

A valid "Risk assessment and management of exposure of healthcare workers in the context of COVID-19" tool developed by WHO was used for the data collection.^9^ Based on the protocol pilot testing was done among 10% of the sample size following the pretesting guideline.^10^ The question was developed on the KoBo Toolbox and was deployed among the study participants as an online questionnaire following the research design guideline for researching on the internet.^11^ The study participants were HCP contributing for COVID-19 management for more than one month. The subject with exposure for one month was real-time monitoring for transmission and mitigating the COVID-19 related infections. The questionnaire was divided into demographic, health care worker information on COVID-19, adherence to IPC during health care interactions, adherence to IPC when performing aerosol-generating procedures (e.g., tracheal intubation, nebulizer treatment, open airway suctioning, collection of sputum, tracheostomy, bronchoscopy, cardiopulmonary resuscitation (CPR), etc.) and accidents with biological materials.

WHO has categorised the risk for COVID-19 as following:^9^

1. Yes, to question 1D & 1E they are considered exposure to the community.
2. Yes, to any question 4A-4D health workers should be considered exposed to COVID-19 virus.
3. High risk: Health care professionals did not respond "always" as recommended to questions 5A1-5G, 6A-6F or responded "Yes" to 7A
4. Low risk: All other answers

In the community setting hospital, most health care professionals were deprived of access to the internet due to which we couldn't reach all participants. Seven subjects didn't complete the form and some information was missed in the questionnaire. All relevant data was entered in individual structured Performa.

Data analysis was done using Statistical Package for the Social Sciences version 23 to calculate the number, percentage, mean, standard deviation. Point estimate at 95% Confidence Interval was calculated.

## RESULTS

Out of 300 health care workers, 109 (36.33%) (30.8541.74 at 95% Confidence Interval) provided direct care to COVID-19 patients. Out of which 61 (55.96%) were female and 48 (44.03%) were male. However, in the age group the active level was at 25-34 with 51 (46.78%) and at least at 45-54 years with 11 (10.09%). Bagmati province had the highest level of involvement in health care followed by Karnali province. With total respondents, 41 (37.61%) were registered nurses, 28 (25.68%) were medical doctors and 36 (33.07%) were paramedics (Table 1).

**Table 1:** Distribution of the respondent by socio demographic characteristic (n= 109).

Participants Details		n (%)
Gender	Male	48 (44.03)
	Female	61 (55.96)
Age Groups (years)	15-24	14 (12.84)
	25-34	51 (46.78)
	35-44	33 (30.27)
	45-54	11 (10.09)
Province	Province-1(Eastern)	19 (17.43)
	Province-2 (Mid-east)	16 (14.84)
	Bagmati Province	27 (24.77)
	Gandaki Province	12 (11.00)
	Province 5 (Mid-west)	16 (14.67)
	Karnali Province	15 (13.76)
	Sudurpachim Province	4 (3.66)
Profession	Medical Doctor	28 (25.68)
	Registered Nurse	41 (37.61)
	Radiology/X-Ray Technician	2 (1.83)
	Ophthalmologist	1 (0.91)
	Paramedics	36 (33.02)
	Public Health	1 (0.91)

Community and travel exposed is null but the exposure from the work is predominately higher based on protocal for risk categorization (Table 2).

**Table 2:** Risk categorization (n = 109).

Details	Yes n (%)	No n (%)	Unknown n (%)
Staying in the same household or classroom environment with confirmed COVID-19	-	109 (100)	-
History of travelling together in close proximity (within 1m) with confirmed COVID-19 patients in any kind of conveyance?	-	109 (100)	-
Provide direct care to a confirmed to COVID-19 patient?	109 (100)	-	-
Face-to-face contact (within 1m) with a confirmed case at a health care facility?	55 (50.45)	49 (45.95)	5 (4.58)
Were you present when any aerosol-generating procedures were performed on the patient?	52 (47.70)	57 (52.29)	-
Did you have direct contact with the environment where the confirmed COVID-19 patient was cared for?	49 (44.95)	60 (55.04)	-
During the period of health care interaction with COVID-19 patients, any episode of accident with biological fluids?	1 (0.91)	108 (99.08)	-

Only 57 (52.3%) have always used the single gloves, one (0.9%) have always used medical masks (Table 3).

**Table 3:** Risk categorization (n= 109).

Details	During a health care interaction with a COVID-19 patient, did you wear personal protective equipment?			
	Always as recommended n (%)	Most of time n (%)	Occasionally n (%)	Rarely n (%)
Single-use gloves	57 (52.29)	29 (26.60)	20 (18.34)	3 (2.75)
Medical mask	1 (0.91)	107 (98.16)	1 (0.91)	-
Face shield or goggles	1 (0.91)	8 (7.33)	15 (13.76)	85 (77.98)
Disposable gown	-	6 (5.50)	3 (2.75)	100 (91.74)
During a health care interaction with the COVID-19 patient, did you remove and replace your PPE according to protocol?	45 (41.28)	51 (46.78)	13 (11.92)	
During a health care interaction with the COVID-19 patient, did you perform hand hygiene before and after touching the COVID-19 patient and immediate environment?	12 (11.00)	40 (36.69)	54 (49.54)	3 (2.75)
During a health care interaction with the COVID-19 patient, did you perform hand hygiene before and after any clean or aseptic procedure was performed?	2 (1.83)	25 (22.94)	15 (13.76)	67 (61.47)
During a health care interaction with the COVID-19 patient, did you perform hand hygiene after exposure to body fluid?	109(100)			
During a health care interaction with the COVID-19 patient, did you perform hand hygiene after touching the patient's surroundings regardless of whether you were wearing gloves?	103(94.49)	6(5.51)		
During a health care interaction with the COVID-19 patient, were high-touch surfaces decontaminated frequently (at least three times daily)?	12 (11.00)	28 (25.69)	65 (59.63)	4 (3.67)

## DISCUSSION

In our study, out of the total health care workers who were directly involved in the care of COVID-19 patient, involvement of registered nurses in the direct care of COVID-19 patient was higher which is similar to the study done by Larribere L, et al.^12^ Out of total participants, 61 (56%) were female which was more than the male 48 (44%). Similar to our study, in Turkey female healthcare professionals are higher than male.^13^ Nursing is a respectable profession in Nepal and is preferred among females only which results in larger female participants.^14^ Kathmandu is the capital and transit point for foreign countries. Studies have revealed that the pandemic is mainly widespread, mostly in places where there is frequent movement of the people.^15^ The government of Nepal has set up COVID special hospitals in each capital of seven provinces.^16^ The first case of COVID-19 in Nepal was seen in Kathmandu from a near-by district.

Out of total participants, no one was exposed to the community. But about 27 (24.8%) participants were exposed to COVID-19 virus from the isolation of 27 (24.8%) followed by laboratory, multi-department and inpatients of hospital settings, it might be due to lack of follow the proper protocol during patient's care. Similar study done in California among the health care professional working in the hospital were found to be highly infected during patient's care following the precautions measures.^17^ The risk of COVID-19 transmission among frontline healthcare professionals worldwide is well known from various studies and the death rate of health care workers in Italy, China, and America due to lack of personal protective equipment (PPE).^18^ Though participants have avoided biological fluids, but other indirect mode of transmission from droplets, fomites, instruments, patients used clothes, were hard to avoidable during the patients' cure. A study done in Delhi for the mode of transmission of COVID-19 have found similar to our findings and have suggested for decreasing human to human contact, daily disinfection and self-daily hygienic measures.^19^

COVID-19 has shown to be transmitted by droplet infection and in our study 52 (47.7%) of participants who responded to their presence in various working units have had exposure to the aerosol generating procedure. Howard, et al. in his study has revealed that aerosol generating procedure in the hospital increased the risk of COVID-19 transmission.^20^ In similar study CDC have no recommendation regarding use of portable purifiers, high efficiency pressure airway (HEPA) for decontamination for isolation settings.^21^ Rooms with negative pressure ventilation with an ante room are ideal to minimise the exposure to aerosol and droplet particle.^4^ HEPA system are an important measure to minimise the mode of transmission the aerosol disease, as the closed space has proven to be easiest mode of transmission of aerosols pathogens.^22^

Removal of dedicated equipment or clothing that are worn by health care professionals for the selfprotection to halt the easy transmission of infectious diseases is important steps to be considered.^23^ In our study only 45 (41.3%) have always removed PPE as recommended and 51 (46.8) have removed most of time. Wrong method of doffing can easily transferred the virus from the PPE to user body.^24^ WHO have suggest various types of PPE based on context of COVID-19 setting, personality and types of diseases.^25^ A similar study in contamination of health care personal in application and removal of PPE have shown that health care personal get infected when they don't follow the safe method, especially while putting on, removal and disposal as this will potentially contaminate the skin and clothing of users.^26,27^

During the health care interaction with COVID cases the use of a medical mask is vital for the preventive measures but in our study, only 1 (0.7%) participants have always used a mask as recommended but 107 (98.2%) have used most of the time. Howard J, et al. in his study have mention that proper and regular application of mask minimise the COVID-19 transmission by 79% in health care settings.^28^ But in contrast to Howard J, et al, a study done among Canadian population have considered three key factors for the mask application which includes the availability of mask, mask population coverage and its effectiveness.^29^ So, due to global shortage of mask our study population were compelled to used it for most of time.

Hand hygiene is also considered as a core element for the prevention and stopping of easy transmission of bacterial and viral infections.^30^ After any aseptic procedures majority of participants have rarely washed their hand 67 (61.6%) though they were using the gloves during the procedure. Other than those working in isolation, none-of the participant groups have reported following hand washing protocols "always". Hand hygiene compliance among the nurses and doctors is universally low, which requires intervention in surgical and ICU.^31^ Similar to our study hand washing among the health care professional working in intensive care unit or any department is found to be poor.^32^ Caregivers often fail to wash their hands although they have positive intention compliance is low in practice.^33^ Studies have suggested that hand washing and alcohol based hand rubbing is one the most effective method for the removal of bacteria and virus.^30^

The risk assessment and management of exposure protocol has recommended the management guideline according to risk categorization. Our study participants are found to be in high-risk categorization and WHO suggestion for the government includes stop interaction with patients if exposure with confirmed cases and keep self for isolation for 14 days; perform the test; psychosocial counselling; provision of compensation and health coverage incase of illness; provide IPC training to health care professionals.

The study has several limitations from participation selection, response bias, short study duration for the data collection, and couldn't include all frontliners despite health care workers in order to find out the prevalence of COVID-19 associated with hand hygiene technique.

## CONCLUSIONS

The prevalence of health care workers who provide direct care to the COVID-19 patient was similar to other studies done in similar settings. About half of the participants were exposed to COVID-19 virus from the hospital setting rather than from the community setting which is similar to the study done in similar setting; it might be due to lack of follow of protocols during COVID-19 patient care. The wide spread of COVID-19 prevalence can be controlled by applying preventive measures especially hand rubbing with alcohol or washing techniques,increasing the manpower, and proper management. The health care workers need to follow the COVID-19 risk management guidelines to minimise the spread of virus and the government needs to prioritise the health care workers safety.
